# Prognostic factors of renal cell cancer in elderly patients: a population-based cohort study

**DOI:** 10.1038/s41598-024-56835-3

**Published:** 2024-03-15

**Authors:** Heini Pajunen, Thea Veitonmäki, Heini Huhtala, Jussi Nikkola, Antti Pöyhönen, Teemu Murtola

**Affiliations:** 1 Faculty of Medicine and Health Technology, Tampere University, Tampere, Finland; 2 Faculty of Social Sciences, Tampere University, Tampere, Finland; 3 Center for Military Medicine, The Finnish Defense Forces, Helsinki, Finland; 4 Department of Urology, TAYS Cancer Center, Tampere, Finland

**Keywords:** Cancer, Renal cancer, Renal cancer

## Abstract

Mortality in renal cell cancer (RCC) is high in the elderly population. Comorbidities have a greater impact on overall prognosis of RCC among elderly patients than in younger patients. All new RCC cases were collected in people over 74 years of age between 1995 and 2018 from the Finnish cancer registry. The comorbidities were identified from the Care Registry for Healthcare. Charlson Comorbidity Index (CCI) was used to evaluate the risk of death based on comorbidities. The overall risk of death was analyzed using the Cox regression model. The risk for RCC death was analyzed using Fine and Gray regression analysis. Individual prognostic role of CCI components was evaluated by adding each component separately into the multivariable Fine and Gray regression model. Using the most prognostic comorbidities we constructed a nomogram to predict RCC mortality. Statistically significant prognostic factors of RCC death were tumor morphology (clear cell, papillary and chromophobe), sex, operative treatment, age, primary tumor extent and CCI. The strongest prognostic factors for overall mortality were tumor extent, tumor morphology and operative treatment. Among the components of CCI, the most important comorbidities predicting mortality were dementia, heart failure and kidney disease. The limitation of this study is that the comorbidities have only been recorded at inpatient and outpatient hospital contacts, which is why the prevalence of comorbidities is probably underestimated. In addition, physical performance status was not available from registry data, but it significantly affects the treatment decisions. RCC mortality is high in the elderly population. Among comorbidities, dementia and heart failure have the greatest impact on the prognosis. Curative treatment in selected elderly patients is efficient and should be considered in patients who can tolerate it and have only limited comorbidities.

## Introduction

Around 3% of all cancers are renal cell cancer (RCC) and the highest incidence of RCC occurs in Western countries^1^. The incidence of RCC increases steadily with age and the peak of incidence is reached at the age of 75 years^2,3^. The annual incidence has increased worldwide and in Europe about 2% in the last two decades until recently. In 2018 approximately 99,200 new RCC cases and 39,100 RCC-related deaths have been reported in the European Union^1^. RCC has the highest mortality rate of all genitourinary cancers and surgery is the only curative treatment for RCC^4^. In one third of patients the disease recurs as metastatic after curative intent treatment. Up to 30% of the patients initially present with metastatic disease^5^. For patients with localized tumors the 5-years survival rate is 88–100%^6^. In the past decades, systemic treatments for metastatic kidney cancer have evolved with the introduction of new drugs such as vascular endothelial growth factor receptor inhibitors, mammalian target of rapamycin inhibitors, and immune checkpoint inhibitors. With these advancements, the median survival of individuals with metastatic kidney cancer has improved to more than four years^7^.

Age, smoking, obesity, hypertension and chronic kidney disease are the known risk factors for RCC according to epidemiological studies^8^. As the population ages and the incidence of known risk factors like hypertension and obesity increases, the incidence of RCC is expected to rise in the upcoming years^9^. The incidence of RCC is twice as high in men as in women^2^.

Despite RCC being the most common cancers with significant mortality in the elderly, factors affecting disease-specific survival among this population are incompletely understood. As the population ages and the incidence of RCC increases this question is becoming ever more relevant^9^. Treatment options in the elderly can be challenging, as comorbidities and frailty become more common along with increasing age. In elderly patients with RCC, functional health is a significant predictor of long-term survival^10^. Often comorbidities may limit life-expectancy more than RCC, indicating conservative treatment of cancer^11^. Surveillance should be considered in elderly and frail patients with asymptomatic renal masses^7^. 15–20% of small renal masses are benign and the majority have low malignant potential^12^. Therefore, it is important to know the factors that influence RCC prognosis in the elderly.

The purpose of this population-based cohort study is to define the key non-RCC related prognostic factors of RCC prognosis in elderly patients who are ≥ 75 years at diagnosis. Furthermore, the aim is to create a risk prognostic nomogram based on non-RCC related factors to facilitate treatment decisions by clinicians. Kutikov et al. has presented a nomogram to quantitate competing causes of mortality in patients with localized RCC^13^. Our nomogram also includes patients with metastasized RCC.

## Materials and methods

### Study cohort

**Figure 1:**
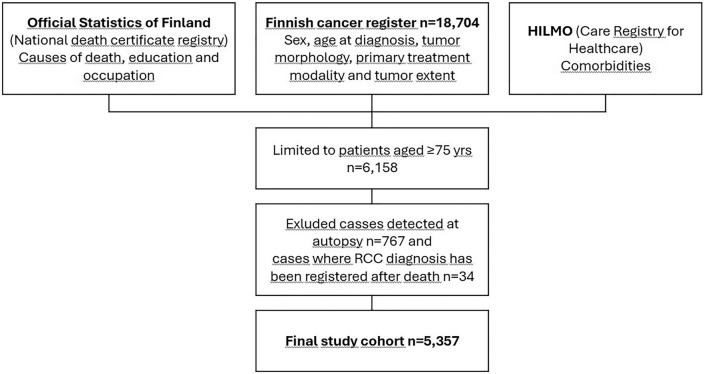
Formation of study cohort.

All newly diagnosed RCC cases were identified in Finland between 1995 and 2018 using the national Finnish Cancer Registry (FCR)^14^. In total 18,704 RCC cases were diagnosed during that time. This study was limited to patients aged ≥ 75 at diagnosis, in a total of 6158 patients. We excluded cases that were detected at autopsy (767 cases) or for unclear reasons the diagnosis was recorded after the day of death (34 cases). Altogether, a cohort of 5357 cases was formed (Fig. 1).

Cancer registry information included sex, age at diagnosis, RCC tumor morphology, primary treatment modality and primary tumor extent. Tumor morphology was divided into four subtypes (clear cell, papillary, chromophobe and missing). Treatment method was categorized as operated (partial nephrectomy or radical nephrectomy) or not operated. Primary tumor extent was categorized as localized (intrarenal or locally advanced) or metastasized.

Information was supplemented with causes of death from the national death certificate registry from Official Statistics of Finland (OSF)^15^. International Classification of Diseases, 9th revision (ICD-9) was used for all diagnoses made in 1995. International Classification of Diseases, 10th revision (ICD-10) coding was used for all diagnoses made between 1996 and 2018. Deaths with ICD-9 code including 1890 and ICD-10 code C64 recorded as the primary cause of death were considered RCC specific deaths. Other causes of death were recorded as non-RCC deaths.

Information about participants' education and occupation was collected from the OSF^15^. Education was divided into secondary education, lowest high-grade, lower university, higher university, research level and unknown. Occupation was divided into physical worker, lower employee and senior officer.

### Information on comorbidities and procedures

The comorbidities were identified from the Care Registry for Healthcare (HILMO) maintained by THL (Finnish institute for health and welfare). From HILMO we collected participants' all diagnoses made at inpatient and out-patient hospital contacts during 1995–2018^16^. We used the Charlson Comorbidity Index (CCI) to evaluate the risk of death based on comorbidities. CCI was calculated as previously described^17^. Comorbidities included complication of diabetes, chronic pulmonary disease, rheumatism, kidney disease, hemiplegia, dementia, heart failure, hepatic impairment, cancer and treatment of metastases. Comorbidities were limited to those that have been diagnosed before RCC.

### Statistical analysis

To analyze the overall risk of death we used Cox regression model. To analyze the risk for RCC-specific deaths in this cohort we used the Fine and Gray model for competing events with adjustment for sex, age at the moment of diagnosis, CCI and socioeconomic factors such as education and occupation. Other reasons for death were treated as competing risks. Time metric was years and months of follow-up since RCC diagnosis. Endpoints were death, emigration or end of 2018, whichever came first. The study population was stratified by age groups (75–79 year, 80–89 year, 90–99 year and 100 year and older) and subgroup analyses were performed for each subgroup.

Individual prognostic role of CCI components was evaluated by adding each component separately into the multivariable Fine and Gray regression model. Beta-coefficients from Cox model was used to create a risk prognostic nomogram for probability of dying of RCC using the “rms” package in R^18^.

As sensitivity analysis we performed a multivariable-adjusted Fine and Gray regression model with further adjustment for RCC clinical characteristics available from the FCR; primary RCC treatment, primary tumor extent and primary tumor morphology.

Analyses were performed using IBM SPSS 27, Stata 17.0 and R version 4.3.

### Ethics statement

Data collection has the ethical permission of the register management authorities. We confirm National Finnish Cancer Registry is publicly available.

## Results

### Population characteristics

The characteristics of the study cohort are described in Table 1. Our cohort consisted of 2976 (55.6%) women and 2381 (44.4%) men. The proportion of women increases in the older age groups. The most common morphology was clear cell cancer (61.8%), then papillary (4.1%) and then chromophobe (1.9%). Morphology data were missing for 32.2% of patients. The number of missing morphologies increased in older age groups, otherwise the distribution of morphology of the tumors was similar in all age groups.

**Table 1:** Characteristics of the study cohort stratified by age at diagnosis.

Characteristics to be considered	75–79 year n 2429		80–89 year n 2599		90–99 year n 324		≥ 100 year n 5	
	n	%	n	%	n	%	n	%
Sex								
Male	1182	48.7	1097	42.2	101	31.2	1	20.0
Female	1247	51.3	1502	57.8	223	68.8	4	80.0
Morphology								
Clear cell	1793	73.8	1463	56.3	51	15.7	1	20.0
Papillary	123	5.1	95	3.7	3	0.9	0	
Chromophobe	62	2.6	39	1.5	2	0.6	0	
Missing	451	18.6	1002	38.6	268	82.7	4	80.0
Primary treatment modality								
Operated	1688	69.5	1271	48.9	38	11.7	0	0
Not operated	741	30.5	1328	51.1	286	88.3	5	100.0
Operative treatment								
Nephrectomy	1482	61.0	1154	44.4	34	10.5	0	0
Partial nephrectomy	224	9.2	127	4.9	4	1.2	0	0
Percutaneous coagulation	15	0.6	22	0.8	2	0.6	0	0
Primary tumor extent								
Localized	811	33.4	682	26.2	36	11.1	1	20.0
Metastasized	609	25.1	668	25.7	87	26.9	2	40.0
Missing	1,009	41.5	1,249	48.1	201	62.0	2	40.0
Status at the end of follow up								
Alive	663	27.3	480	18.5	14	4.3	0	0
Death (all causes)	1766	72.7	2119	81.5	310	95.7	5	100.0
RCC deaths	960	39.5	1257	48.4	233	71.9	4	80.0
Non-RCC deaths	621	25.6	589	22.7	36	11.1	0	0
Charlson index; median (IQR)	2	2–3	2	2–3	2	2–4	2	2–3
Acquired renal dysfunction	136	5.6	143	5.5	15	4.6	0	0

More than half of the patients (55.9%) in the cohort underwent surgery. The proportion of those treated with surgery decreased in older age groups and none of the patients in the oldest age group underwent surgery. The majority of those treated operatively underwent nephrectomy. Only 6.6% underwent partial nephrectomy. Percutaneous ablative therapy was performed for 39 patients (0.7%). At the time of diagnosis, the disease was local in 28.6% and metastasized in 25.5%. The data on tumor extent were missing for 45.9% of patients. The primary tumor extent was similar in all age groups. The median Charlson index was 2 and it remained the same in all age groups. During the follow-up period 5% of patients were diagnosed with kidney deficiency.

### Renal cancer specific survival and overall survival by age-group

**Figure 2:**
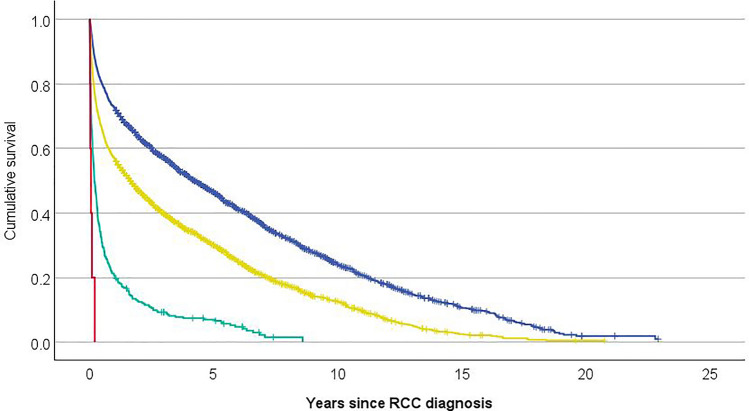
Overall survival among elderly Finnish renal cancer patients by age-group. Blue dashed line 75–79 year, yellow dashed line 80–89 year, green dashed line 90–99 year, red dashed line ≥ 100 year.

In total 45.8% of patients died of RCC during median follow-up of 2 years. In 55.1% RCC was the primary or underlying cause of death and 23.3% died of another cause than RCC resulting in an overall mortality rate of 78.4%. The median time from diagnosis to death for patients who died during follow-up was 13 months. Overall survival among elderly Finnish RCC patients by age groups is shown in Fig. 2. Mortality from RCC and overall mortality increased in older age groups compared to cases diagnosed at 75–79 years of age.

### Prognostic factors

In the Fine and Gray model for competing risk analysis independent and statistically significant prognostic factors of RCC death were tumor morphology, sex, operative treatment, age, primary tumor extent and CCI (Table 2). Primary tumor extent, tumor morphology and operative treatment were strongly prognostic in all age groups. Age, sex and CCI were weaker prognostic factors. Their effect remained more or less the same in all age groups, although not statistically significant in all age groups.

**Table 2:** Risks of RCC death under the presence of competing risks. Sub hazard ratios (sHR) with 95% Cis in multivariate adjusted model.

Characteristic to be considered	RCC deaths n	sHR	95% CI
Age group			
75–95 year	960	1	
80–89 year	1257	0.99	0.91–1.09
90–99 year	233	1.12	0.94–1.34
≥ 100 year	4	1.83	0.37–9.23
Tumor morphology			
Clear cell	1029	1	
Papillary	40	0.57	0.42–0.77
Chromophobe	6	0.20	0.09–0.44
Missing	1379	2.40	2.13–2.70
Sex			
Male	1083	1	
Female	1371	0.97	0.88–1.05
Primary treatment modality			
Not operated	1659	1	
Operated	795	0.49	0.44–0.55
Pre dg CCI		0.96	0.92–1.00
Education			
Secondary education	346	1	
Lowest high-grade	130	0.99	0.80–1.22
Lower university	85	0.87	0.67–1.13
Higher university	49	0.99	0.74–1.33
Researcher level	1	0.19	0.02–1.50
Unknown	1843	1.03	0.91–1.16
Occupation			
Senior officer	16	1	
Lower employee	36	0.66	0.38–1.14
Physical worker	38	0.66	0.38–1.14
Unknown	2364	0.77	0.49–1.21
Primary tumor extent			
Localized	350	1	
Metastasized	1088	3.67	3.23–4.17
Missing	1016	1.86	1.65–2.11

When considering overall mortality, the most influential factors were tumor extent, morphology, and surgical treatment (Supplementary Table 1). Interestingly, biological sex played a more prominent role in predicting the overall risk of death compared to its role in predicting death from RCC. CCI also played a role in predicting overall mortality, and its effect persisted across different age groups.

**Figure 3:**
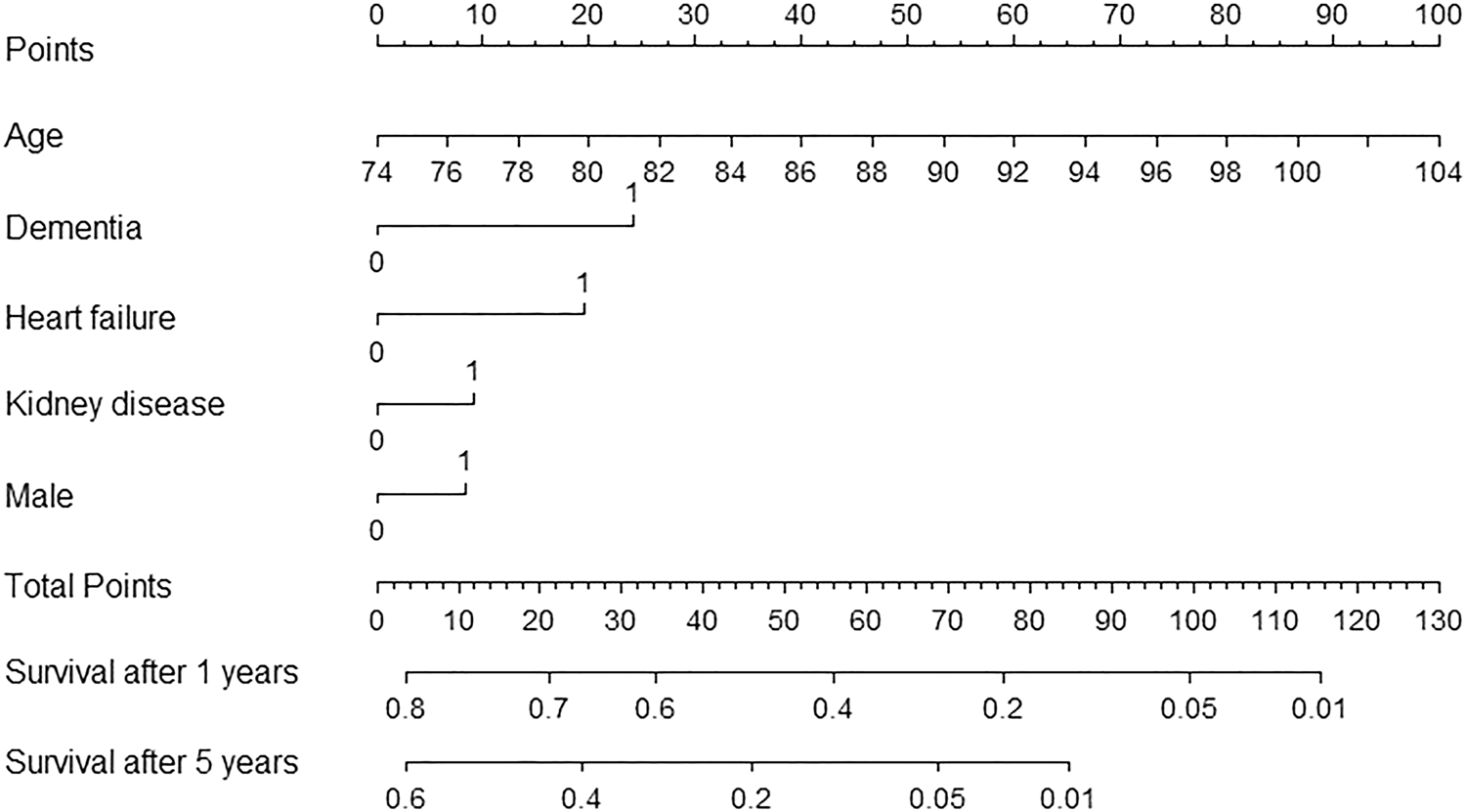
A nomogram to predict the risk of renal cancer death among elderly renal cancer patients, including age, sex and the most prognostic comorbidities.

Analyzing the components of the CCI, several conditions stood out as significant prognostic factors. These included dementia, heart failure and kidney disease. Additionally, age at diagnosis and male sex were prognostic factors (Supplementary Table 2). A nomogram was created based on age, sex, clinical characteristics of the tumor, and the mentioned prognostic conditions. This nomogram demonstrated an accuracy of 0.64 in predicting mortality from kidney cancer among elderly patients (Fig. 3).

### Sensitivity analysis

For a sensitivity analysis we broadened the definition of RCC death to include deaths where RCC was recorded among the contributory causes. The risk factors for this RCC-related mortality remained the same as in the main analysis, where RCC was required to be the primary cause of death (Supplementary Table 3).

In addition, we performed a sensitivity analysis limited to participants with information on tumor extent available. In this subgroup analysis, age and CCI were even stronger prognostic factors, especially in people ≥ 90 years old.

## Discussion

In a population-based cohort of elderly RCC patients, we found the RCC specific mortality to be high, as stated in previous publications^19^. Surgical treatment and patient age were the strongest prognostic factors, whereas dementia and heart failure were the comorbidities that had the greatest impact on survival. Similar results were found in a previous study^20,21^. Therefore, these factors should be taken into account when considering treatment in elderly RCC patients.

During the follow-up period, 45.8% of patients died of RCC. Other studies have also found that although RCC treatment results have improved, the mortality rate of elderly people from RCC remains high^22^. With the increasing average life expectancy, elderly people are expected to live longer in the future. Thus, actively treating RCC in selected elderly patients can improve the overall survival from localized RCC in this population. Other studies have also shown that elderly patients benefit from operative treatment^3^. Operative treatment is the only curative treatment for RCC. At the beginning of the study period, nephrectomy was the standard procedure and partial nephrectomy only became more common at the end of the study, which explains the small number of partial nephrectomies. Also, mini-invasive surgery became more common in Finland after the 2010s. Operative treatment is one of the strongest predictors of RCC and overall survival and therefore should be offered to suitable elderly patients with limited comorbidities and fit enough to tolerate surgery.

Our nomogram helps to evaluate risk of RCC-related mortality while taking into account both clinical factors and comorbidities, which facilitates treatment decisions in elderly RCC patients. The nomogram was made with a population-based cohort covering all newly diagnosed RCC cases in Finland. Thus, the nomogram is likely generalizable to Nordic populations or possibly other Caucasian populations. Accuracy of the nomogram in other ethnicities will require validation.

In our cohort 55.6% of patients were women. The large proportion of women is explained by longer average life expectancy; consequently, the proportion of women is higher in the Finnish elderly population.

In previous studies surgery and age were the biggest prognostic factors of RCC in the elderly. Tumor size, Fuhrman grade, morphology, number of tumors, T-score and marital status also affected overall survival^22^. We did not have all these previously studied risk factors available. However, as elderly and frail patients are commonly excluded from clinical trials it is important to do population-based studies to characterize predictors of survival for RCC in this population.

The strength of the study is the large data that covers all Finnish RCC patients and their comorbidities from 1995 to 2018. The information was available from comprehensive and reliable cancer registries. As the study cohort covers all elderly RCC cases in Finland in the study period, the study gives a comprehensive review of RCC care in the target group.

This study has limitations. The comorbidities have only been recorded at inpatient and outpatient hospital contacts, which is why the prevalence of comorbidities is probably underestimated. This likely creates bias towards the null, which explains why CCI is not a stronger prognostic factor. Cancer Registry data was missing the tumor extent for 45.9% of patients, which is a major limitation of the study. This also has likely caused bias towards the null, as the prognostic associations were stronger in sensitivity analysis limited to participants with information on tumor extent. Results need to be confirmed in other studies with more comprehensive knowledge on tumor extent. Furthermore, information on physical performance status, which significantly affects the treatment decisions, was not available from registry data. Also, we were not able to evaluate the effect of operative treatment on the patient's need for home care or institutionalization. In our cohort 55.9% underwent operative treatment. The large number of conservatively treated patients explains why a large group of the patients lacks tumor morphological data.

## Conclusions

RCC mortality is high in the elderly population. Of the co-morbidities, dementia and heart failure have the greatest impact on the prognosis. Curative treatment in selected patients aged over 74 years is efficient and should be considered in patients who tolerate it and have limited comorbidities.

### Supplementary Information

Supplementary Tables.

## Data Availability

De-identified study data and materials are available from the authors at request. The request of the data can be sent to Teemu Murtola. Contact information: Teemu Murtola, email: Teemu.Murtola@tuni.fi, Faculty of Medicine and Health Technology, Tampere University, Tampere, Finland.
