# Supplementary material for: Prognostic factors of renal cell cancer in elderly patients: a population-based cohort study
Source: Sci Rep. 2024 Mar 15;14:6295. doi: 10.1038/s41598-024-56835-3 (PMC10942969; doi:10.1038/s41598-024-56835-3)
Supplement: Supplementary file 1 — Supplementary Tables. [file 41598_2024_56835_MOESM1_ESM.doc]

# Prognostic factors of renal cell cancer in elderly patients – a population-based cohort study

Heini Pajunen¹*, Thea Veitonmäki¹, Heini Huhtala², Jussi Nikkola¹, Antti Pöyhönen³, Teemu Murtola¹⁴

1 Faculty of Medicine and Health Technology, Tampere University, Tampere, Finland

2 Faculty of Social Sciences, Tampere University, Tampere, Finland

3 Center for Military Medicine, The Finnish Defense Forces, Helsinki, Finland

4 Department of Urology, TAYS Cancer Center, Tampere, Finland

*Correspondence: Heini Pajunen (email: [heini.e.pajunen@gmail.com](mailto:heini.e.pajunen@gmail.com)).

Correspondence: Faculty of Medicine and Health Technology, Tampere University, Tampere, Finland (telephone: +3583749364, email: heini.e.pajunen@gmail.com).

**Supplementary table 1 - Predictors of overall risk of death. HR multivariate adjusted 95% CI.**

|  | All 75 yr and older  n5357 | |  | 75-79 yr  n 2429 | |  | 80-89 yr  n 2599 | |  | 90-99 yr  n 324 | |
| --- | --- | --- | --- | --- | --- | --- | --- | --- | --- | --- | --- |
| Characteristics | n (cases/RCC deaths) | HR (95% CI) | | | HR (95% CI) | | | HR (95% CI) | | | HR (95% CI) |
| Tumor morphology | |  | | |  | | |  | | |  |
| Clear cell | 3,308/2,353 | 1 | | | 1 | | | 1 | | | 1 |
| Papillary | 221/109 | 0.75 (0.62–0.91) | | | 0.70 (0.53–0.92) | | | 0.82 (0.62–1.08) | | | 1.99 (0.57–6.91) |
| Chromophobe | 103/39 | 0.60 (0.44–0.82) | | | 0.71 (0.46–1.09) | | | 0.53 (0.32–0.87) | | | 0.85 (0.11–6.36) |
| Gender | |  | | |  | | |  | | |  |
| Male | 2,381/1,900 | 1 | | | 1 | | | 1 | | | 1 |
| Female | 2,976/2,300 | 0.79 (0.74–0.84) | | | 0.74 (0.67–0.81) | | | 0.82 (0.75–0.90) | | | 0.80 (0.63–1.03) |
| Primary RCC treatment | |  | | |  | | |  | | |  |
| Not operated | 2,360/2,224 | 1 | | | 1 | | | 1 | | | 1 |
| Operated | 2,997/1,976 | 0.50 (0.46–0.55) | | | 0.47 (0.42–0.53) | | | 0.55 (0.48–0.62) | | | 0.46 (0.26–0.81) |
| Age at diagnosis |  | 1.05 (1.04–1.06) | | | 1.05 (1.02–1.09) | | | 1.05 (1.03–1.06) | | | 1.02 (0.97–1.08) |
| Pre dg CCI |  | 1.12 (1.10–1.15) | | | 1.17 (1.12–1.21) | | | 1.11 (1.06–1.15) | | | 1.09 (1.00–1.19) |
| Education | |  | | |  | | |  | | |  |
| Secondary | 816/591 | 1 | | | 1 | | | 1 | | | 1 |
| Higher | 684/469 | 0.93 (0.82–1.05) | | | 0.93 (0.77–1.12) | | | 0.84 (0.71–1.01) | | | 0.97 (0.60–1.58) |
| Occupation | |  | | |  | | |  | | |  |
| Senior officer | 47/19 | 1 | | | 1 | | | 1 | | | 1 |
| Lower employee | 115/57 | 1.08 (0.64–1.82) | | | 1.26 (0.64–2.47) | | | 1.12 (.47–2.66) | | | 0 |
| Physical worker | 109/60 | 1.21 (0.72–2.03) | | | 1.74 (0.89–3.39) | | | 0.71 (0.28–1.76) | | | 0 |
| Primary tumor extent | |  | | |  | | |  | | |  |
| Localized | 1,530/1,259 | 1 | | | 1 | | | 1 | | | 1 |
| Metastasized | 1,366/1,288 | 2.06 (1.89–2.24) | | | 2.41 (2.12–2.47) | | | 1.93 (1.70–2.19) | | | 2.17 (1.40–3.36) |

RCC = Renal Cell Carcinoma; Pre dg CCI = Charlson Comorbidity Index calculated based on conditions diagnosed before RCC

**Supplementary table 2 – Prognostic role of separate CCI components any compared to none stratified by age group.**

| Component of CCI at time of RCC diagnosis | all **≥**75 yr |  | 75–79 yr |  | 80–89 yr |  | 90–99 yr |
| --- | --- | --- | --- | --- | --- | --- | --- |
|  | HR (95% CI) |  | HR (95% CI) |  | HR (95% CI) |  | HR (95% CI) |
| Complication of diabetes | 1.19 (1.07–1.32) |  | 1.30 (1.10–1.55) |  | 1.14 (0.99–1.31) |  | 1.34 (0.91–1.97) |
| Chronic pulmonary disease | 1.13 (1.01–1.27) |  | 1.20 (1.01–1.43) |  | 1.21 (1.03–1.42) |  | 0.78 (0.48–1.26) |
| Rheumatism | 1.02 (0.84–1.25) |  | 1.10 (0.82–1.48) |  | 0.99 (0.75–1.32) |  | 2.09 (0.97–4.48) |
| Kidney disease | 1.39 (1.09–1.78) |  | 1.09 (0.68–1.75) |  | 1.38 (0.99–1.94) |  | 1.45 (0.81–2.59) |
| Hemiplegia | 1.41 (0.83–2.39) |  | 6.35 (2.37–17.03) |  | 1.05 (0.57–1.96) |  | 0 |
| Dementia | 2.25 (1.94–2.62) |  | 2.17 (1.61–2.94) |  | 2.10 (1.73–2.54) |  | 1.33 (0.87–2.03) |
| Heart failure | 1.78 (1.61–1.97) |  | 1.76 (1.47–2.11) |  | 1.59 (1.39–1.82) |  | 1.46 (1.08–1.98) |
| Hepatic impairment | 0.92 (0.62–1.37) |  | 1.30 (0.78–2.17) |  | 0.86 (0.46–1.63) |  | 0 |
| Treatment of metastases | 1.45 (0.87–2.42) |  | 1.72 (0.91–3.25) |  | 1.74 (0.64–4.76) |  | 8.56 (1.17–62.33) |
| Other cancer | 1.10 (1.00–1.21) |  | 1.05 (0.90–1.23) |  | 1.06 (0.93–1.20) |  | 1.22 (0.89–1.65) |

CCI = Charlson Comorbidity Index; RCC = Renal Cell Cancer

**Supplementary table 3 –** **Predictors of extended RCC death. HR multivariate adjusted 95% CI.**

|  | all **≥**75 yr | |  | | 75–79 yr |  | | 80–89 yr |  | 90–99 yr |
| --- | --- | --- | --- | --- | --- | --- | --- | --- | --- | --- |
| Characteristics | n (cases/RCC deaths) | HR (95% CI) | | HR (95% CI) | | | HR (95% CI) | | | HR (95% CI) |
| Tumor morphology | |  | |  | | |  | | |  |
| Clear cell | 3,308/1,320 | 1 | | 1 | | | 1 | | | 1 |
| Papillary | 221/59 | 0.66 (0.51–0.86) | | 0.56 (0.38–0.83) | | | 0.80 (0.56–1.15) | | | 0.90 (0.12–7.02) |
| Chromophobe | 103/10 | 0.24 (0.13–0.45) | | 0.29 (0.31–0.66) | | | 0.22 (0.08–0.60) | | | 0 |
| Gender | |  | |  | | |  | | |  |
| Male | 2,381/1318 | 1 | | 1 | | | 1 | | | 1 |
| Female | 2,976/1,636 | 0.86 (0.80–0.93) | | 0.85 (0.75–0.96) | | | 0.87 (0.78–0.96) | | | 0.79 (0.60–1.03) |
| Primary RCC treatment | |  | |  | | |  | | |  |
| Not operated | 2,360/1,906 | 1 | | 1 | | | 1 | | | 1 |
| Operated | 2,997/1,048 | 0.43 (0.39–0.48) | | 0.42 (0.36–0.49) | | | 0.46 (0.39–0.54) | | | 0.42 (0.22–0.81) |
| Age at diagnosis |  | 1.04 (1.03–1.05) | | 1.03 (0.99–1.07) | | | 1.03 (1.01–1.05) | | | 1.01 (0.95–1.07) |
| Pre dg CCI |  | 1.08 (1.05–1.11) | | 1.10 (1.05–1.16) | | | 1.07 (1.02–1.11) | | | 1.10 (1.00–1.22) |
| Education | |  | |  | | |  | | |  |
| Secondary | 816/407 | 1 | | 1 | | | 1 | | | 1 |
| Higher | 684/307 | 0.91 (0.78–1.06) | | 1.00 (0.79–1.26) | | | 0.78 (0.63–0.97) | | | 1.11 (0.67–1.84) |
| Occupation | |  | |  | | |  | | |  |
| Senior officer | 47/17 | 1 | | 1 | | | 1 | | | 1 |
| Lower employee | 115/40 | 0.77 (0.43–1.38) | | 0.92 (0.44–1.90) | | | 0.71 (0.27–1.89) | | | 0 |
| Physical worker | 109/47 | 0.89 (0.51–1.56) | | 1.27 (0.62–2.60) | | | 0.52 (0.19–1.41) | | | 0 |
| Primary tumor extent | |  | |  | | |  | | |  |
| Localized | 1,530/540 | 1 | | 1 | | | 1 | | | 1 |
| Metastasized | 1,366/1,159 | 3.03 (2.71–3.39) | | 3.70 (3.13–4.36) | | | 2.78 (2.36–3.27) | | | 2.83 (1.71–4.68) |

RCC = Renal Cell Cancer; Extended renal cancer death = RCC as the primary or underlying cause of death; Pre dg CCI = Charlson Comorbidity Index calculated based on conditions diagnosed before RCC
